# Anticipatory Heart Rate Before Moderate- and Vigorous-Intensity Exercise Among Sedentary and Physically Active Young Adult Males

**DOI:** 10.7759/cureus.49707

**Published:** 2023-11-30

**Authors:** Chinmay Das, Baidyanath Mishra, Shaikat Mondal, Himel Mondal

**Affiliations:** 1 Physiology, Saheed Laxman Nayak Medical College and Hospital, Koraput, IND; 2 Physiology, Sri Jagannath Medical College and Hospital, Puri, IND; 3 Physiology, Raiganj Government Medical College and Hospital, Raiganj, IND; 4 Physiology, All India Institute of Medical Sciences, Deoghar, Deoghar, IND

**Keywords:** parasympathetic stimulation, sympathetic response, young adults, sedentary, oximetry, heart rate, jogging, exercise response, exercise, autonomic control

## Abstract

Background

Anticipatory heart rate (HR) refers to an increase in HR that occurs in anticipation of a future event or activity. The anticipatory heart rate (HR) response before exercise is an important physiological indicator of exercise readiness. This study aimed to compare the anticipatory HR changes between sedentary and physically active young adult males during moderate- and vigorous-intensity exercise. Understanding these anticipatory heart rate patterns can provide insights into the physiological adaptations and cardiovascular health of individuals with varying physical activity levels.

Materials and methods

A total of 60 young adult males, comprising sedentary (n = 30) and physically active individuals (n = 30), participated in this study. A brisk walking for a distance of 50 m was considered moderate intensity and one minute of spot jogging at maximum effort with verbal encouragement was considered vigorous intensity exercise. The HR was recorded at baseline, just before the exercise, and on each minute up to 5 minutes after the exercise.

Results

The study involved 30 physically active young adult males (mean age 20.23 ± 1.43 years) and 30 sedentary adult males (mean age 20.07 ± 1.17 years). In physically active young adults, the resting HR was 76.4±10.89 bpm and just before starting moderate-intensity exercise, it was 78.83±12.98 bpm, paired t-test P = 0.22. The HR just before vigorous-intensity exercise was 80.83±11.18 bpm (paired t-test P = 0.03). In sedentary young adults, the resting HR was 82.23±12.69 bpm and just before starting moderate-intensity exercise, it was 90.13±18.69 bpm, paired t-test P = 0.0008. The HR just before vigorous-intensity exercise was 91.7±15.04 bpm (paired t-test P <0.0001).

Conclusion

Physically active young adults did not exhibit a significant increase in anticipatory HR before moderate-intensity exercise. However, sedentary individuals exhibit a significant anticipatory HR response. Before vigorous-intensity exercise, both exhibited significant increments in HR. The result highlights the importance of considering the anticipatory HR response as a potential marker of cardiovascular health and exercise readiness.

## Introduction

Exercise anticipation elicits a range of cognitive (e.g., attention and arousal), somatic (e.g., skeletal muscle tension), and visceral (e.g., autonomic response) changes, preparing the body for physical activity. These changes primarily occur due to signals from the motor cortex to the brainstem cardiorespiratory centres [1]. The autonomic nervous system (ANS), responsible for regulating blood pressure, heart rate (HR), and peripheral vascular resistance, plays a crucial role in maintaining homeostasis. The balance between sympathetic and parasympathetic outflow is pivotal for appropriate cardiovascular responses at rest and during exercise [2].

Prolonged sedentary lifestyles have been found to disrupt this balance, leading to lower parasympathetic activity in sedentary individuals [3]. Conversely, higher levels of physical activity have been reported to promote increased parasympathetic activity in the heart [4].

The anticipatory increase in HR provides valuable insights into quantifying sympathetic and parasympathetic activity. During the anticipatory phase before exercise, the sympathetic nervous system becomes activated, leading to an increase in HR [5]. This sympathetic activation prepares the body for the upcoming physical activity by increasing cardiovascular output and mobilizing energy resources. The anticipatory increase in HR reflects the sympathetic dominance in response to the cognitive and somatic cues associated with exercise anticipation. On the other hand, parasympathetic activity, mediated by the vagus nerve, acts to oppose sympathetic activity and promote cardiovascular recovery and relaxation [6]. Hence, we can infer differences in sympathetic and parasympathetic activity by comparing the anticipatory HR response between sedentary and physically active individuals [7, 8].

Miyamoto et al. studied eleven healthy men and concluded that anticipatory cardiorespiratory adjustments, controlled by the higher brain, occur before starting exercise and contribute to reducing the time delay in circulatory response [6]. These adjustments are believed to enhance performance during high-intensity exercise in humans.

Koppula et al. studied twenty-nine healthy males and found that anticipating exercise leads to changes in cardiorespiratory coherence, similar to those observed during the actual exercise [9]. These findings indicate that the control mechanisms involving the cardiovascular, respiratory, and autonomic systems before movement are influenced by the specific task. Furthermore, these anticipatory changes enable the prediction of subsequent motor performance.

However, no previous study ascertained the comparative anticipatory HR between sedentary and physically active young males in different grades of exercise. Hence, our study aimed to investigate the differences in anticipatory HR (i.e., the pre-exercise increase in HR induced by thoughts about the exercise) among sedentary and physically active young adult males in moderate- and vigorous-intensity exercise.

## Materials and methods

Type and setting

This was a cross-sectional observational study conducted in a medical college laboratory situated in the eastern zone of India. The area where the study was conducted is hilly and had an average of 870 m elevation. The study was conducted from January to March 2023.

Ethics

The study protocol was approved by the Institutional Ethics Committee of Saheed Laxman Nayak Medical College and Hospital. All the research participants were recruited after obtaining written informed consent. The subjects were first briefed about the study protocol in detail and were recruited if they wanted to participate voluntarily.

Sample

The study was conducted with a convenience sample. Any apparently healthy young adult male (age 18-26 years) was eligible to enrol in the study. Purposively we recruited 30 physically active and 30 sedentary males. For the sedentary group, the individual must not practice any additional walking, playing, or exercise except for their daily routine work. For the physically active group, individuals doing exercise in any form like walking, cycling, wheeling, doing sports, or active recreation at least 1 hour a day and 6 days a week for at least the last 3 months were recruited. Physically active subjects were recruited from the institutional gymnasium.

Sample size

A previous study has reported a mean resting HR of 72 and anticipatory HR of 138 with a standard deviation of the outcome variable of 28 [10]. With this input, alpha = 0.05, and power of the study 95%, equal distribution of subjects in each group, and 20% additional subjects, the minimum sample size was 11 according to the formula: N = [(1/q1 + 1/q2) S^2^ (Zα + Zβ)^2^] ÷ E^2^ where q1 and q2 denote the proportion of subjects in each group (keep 50 in if equal numbers in each group are desired), S is the standard deviation of the outcome variable, Zα denotes the standard normal deviate for α, Zβ denotes the standard normal deviate for β, E is the Effect size calculated from the difference of the mean of two groups. However, to get a scalable sample for statistical comparison, we decided to include 30 sedentary and 30 physically active participants. This satisfies the minimum sample size and increases the power of the study.

Instruments

For HR (bpm) measurement, a portable pulse oximeter - BPL SmartOxy (BPL Medical Technologies, Bengaluru, India) - was used. Omron's automated blood pressure monitor - Omron HEM 7120 (Omron Healthcare Technologies, Kyoto, Japan) - was used for measuring arterial blood pressure. For weight measurement, we used a digital weighing scale with 100 gm sensitivity, and for height and waist circumference, we used a fibreglass measuring tape.

Exercise plan

Moderate- and vigorous-intensity exercise program was fixed according to the protocol proposed and validated by Mondal et al. [11]. A brisk walking for a distance of 50 m is categorized as moderate-intensity exercise and one minute of spot jogging at maximum effort with verbal encouragement was categorized as vigorous-intensity exercise.

Measurements

**Figure 1:**
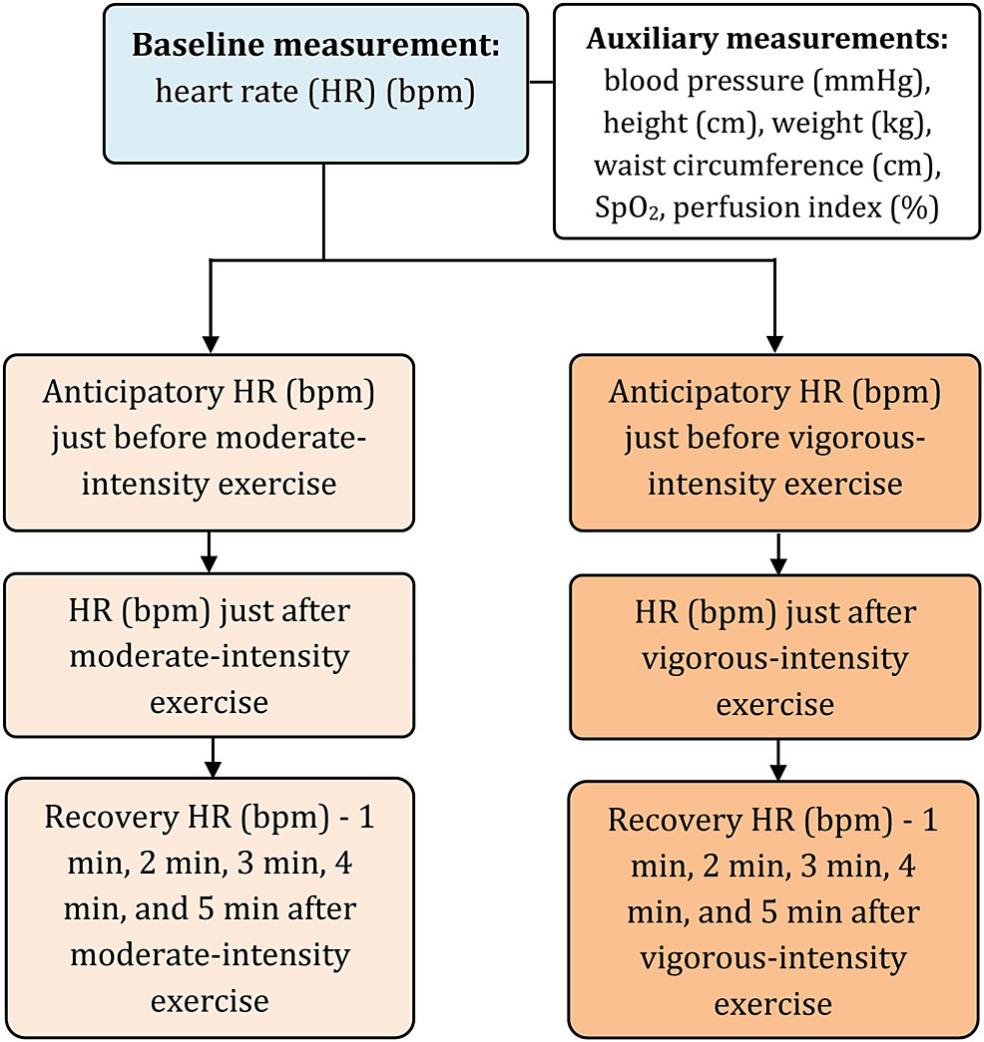
Measurement flow of heart rate and other parameters bpm: beats per minute; HR: heart rate; SpO2: peripheral oxygen saturation

All the measurements were done in a controlled environment with 25°C and 50% humidity in the laboratory. There were same-sex attendants during all the measurements. The blood pressure was measured by a single expert observer to reduce any inter-observer variation. The measurement steps are presented in a flow chart in Figure 1.

Statistical analysis

After data collection, the data were first expressed in descriptive statistics. Then it was tested for distribution (the Shapiro-Wilk test) to check if the data is normally distributed or not. According to the findings, further analysis was chosen. Resting HR and anticipatory HR before exercise was tested by paired t-test. Data between the sedentary and active groups were tested by either an unpaired t-test or a Mann-Whitney U test (non-parametric test). The change in HR in resting, the anticipation of exercise, and HR during recovery was tested statistically through Analysis of Variance (ANOVA) with post hoc analysis. We used Microsoft Excel 2010 (Microsoft Corporation, Redmond, USA) for calculating mean, standard deviation, median, interquartile range, etc. as descriptive statistical tests and GraphPad Prism 6.01 (GraphPad Software, La Jolla, USA) for inferential statistical tests.

## Results

A total of 30 physically active young adult males with a mean age of 20.23 ± 1.43 years and 30 sedentary adult males with a mean age of 20.07 ± 1.17 years participated in the study. The age, anthropometric, and cardiovascular parameters are shown in Table 1.

**Table 1:** Age, anthropometric, and cardiovascular parameters in physically active and sedentary young adult males *Statistically significant p-value of unpaired t-test BMI: Body mass index, SpO2: Saturation of peripheral oxygen

Parameter	Physically active (n = 30)	Sedentary (n = 30)	p-value
Age (years)	20.23±1.43	20.07±1.17	0.62
Height (cm)	173.7±5.07	171.63±4.43	0.09
Weight (kg)	67.67±8.29	73.92±11.56	0.02*
BMI (kg/m^2^)	22.43±2.59	25.02±3.18	0.001*
Waist circumference (cm)	78.65±6.18	84.72±10.46	0.008*
Systolic blood pressure (mmHg)	124.23±9.06	120.13±11.02	0.12
Diastolic blood pressure (mmHg)	79.47±8.46	81.47±9.14	0.38
SpO_2_ (%)	97.03±1.6	96.67±1.79	0.41
Perfusion index (%)	4.37±2.37	4.26±3.08	0.88

Physically active young adult males had significantl lower weight, BMI, and waist circumference compared to their sedentary counterparts. However, there were no significant differences in height, blood pressure, oxygen saturation, and perfusion index between the two groups.

**Figure 2:**
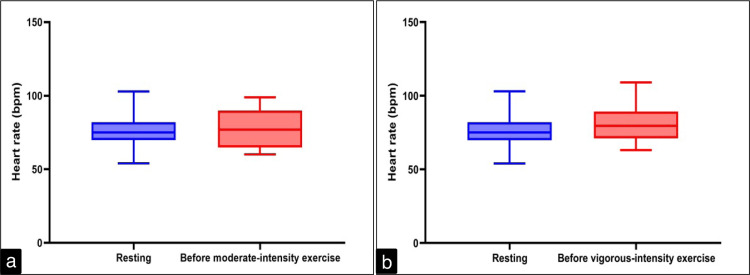
Heart rate in resting and just before moderate-intensity exercise (a) and vigorous-intensity exercise (b) in physically active young adult males bpm: beats per minute

In physically active young adults, the resting HR was 76.4±10.89 bpm, and just before starting moderate-intensity exercise, it was 78.83±12.98 bpm, paired t-test P = 0.22 (Figure 2a). The HR just before vigorous-intensity exercise was 80.83±11.18 bpm (paired t-test P = 0.03, Figure 2b).

**Figure 3:**
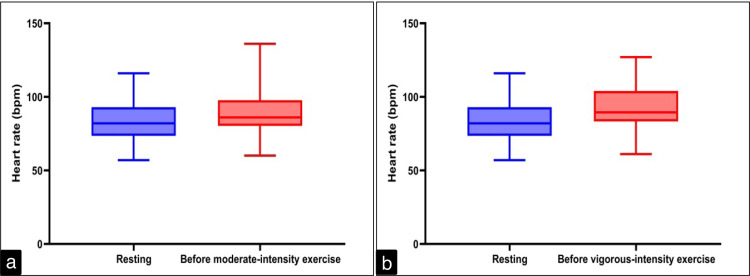
Heart rate in resting and just before moderate-intensity exercise (a) and vigorous-intensity exercise (b) in sedentary young adult males bpm: beats per minute

In sedentary young adults, the resting HR was 82.23±12.69 bpm, and just before starting moderate-intensity exercise, it was 90.13±18.69 bpm, paired t-test P = 0.0008 (Figure 3a). The HR just before vigorous-intensity exercise was 91.7±15.04 bpm (paired t-test P <0.0001, Figure 3b)

**Figure 4:**
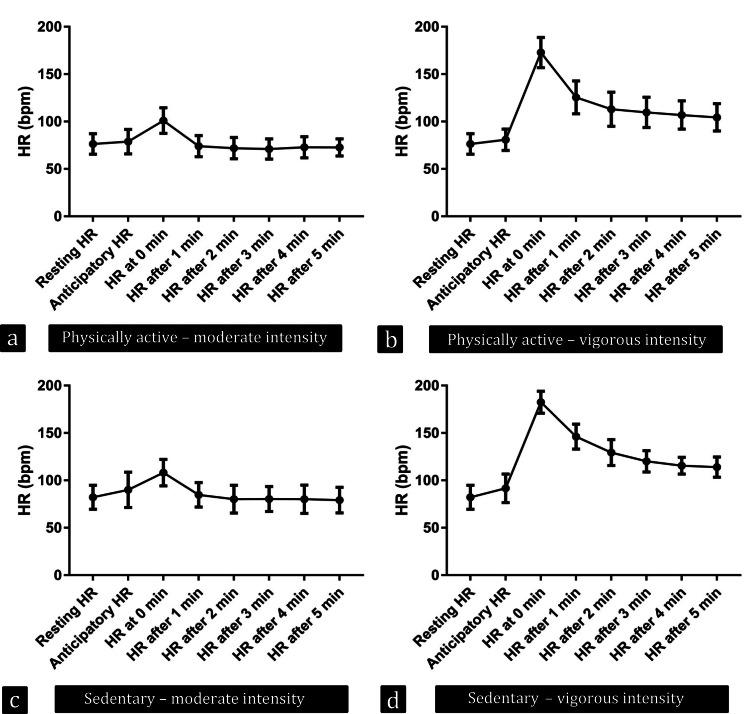
Resting, anticipatory, and recovery heart rate in the studied participants Resting, anticipatory, and recovery heart rate in (a) physically active participants in moderate-intensity exercise, (b) physically active participants in vigorous-intensity exercise, (c) sedentary participants in moderate-intensity exercise, and (d) sedentary participants in vigorous-intensity exercise HR: Heart rate, bpm: beats per minute

In the physically active group, there was an overall significant change in resting HR in anticipation, during, and after moderate exercise, ANOVA F = 56.64, p-value <0.0001 (Figure 4a).

However, there was no significant change between resting and anticipatory HR in the post hoc test (adjusted p-value = 0.91). The overall significance in ANOVA was due to a sharp rise in HR immediately after exercise.

Similarly, although there was a significant overall change in resting HR in anticipation of vigorous exercise and at the end and during recovery in the physically active group, ANOVA F = 118.1, p-value <0.0001 (Figure 4b), there was no significant change between resting and anticipatory HR in the post hoc test (adjusted p-value = 0.95).

In sedentary young adult males, there was an overall significant change of resting HR in anticipation of moderate exercise, and at the end and after the exercise, ANOVA F = 43.53, p-value <0.0001 (Figure 4c). There was a significant change between resting and anticipatory HR in the post hoc test (adjusted p-value = 0.015).

Similarly, there was an overall significant change in resting HR in anticipation of moderate exercise, and at the end and after the exercise, ANOVA F = 481.7, p-value <0.0001 (Figure 4d). There was a significant change between resting and anticipatory HR in the post hoc test (adjusted p-value = 0.002).

The anticipatory and post-exercise HR in moderate-intensity exercise between active and sedentary males is shown in Table 2.

**Table 2:** Anticipatory and post-exercise heart rate in moderate-intensity exercise ^*^Statistically significant p-value of unpaired t-test ^†^p-value of Mann-Whitney U test HR: Heart rate, Δ_anticipatory-rest_: Difference of anticipatory and resting heart rate

Parameter	Physically active (n = 30)	Sedentary (n = 30)	p-value
Resting HR	76.4±10.89	82.23±12.69	0.06
Anticipatory HR	78.83±12.98	90.13±18.69	0.009*
Δ _anticipatory-rest_	2.4±10.69	7.9±11.5	0.067†
HR immediately after exercise	101.03±13.48	108.23±14.03	0.047*
HR after 1 min	74.07±11.17	84.8±12.87	0.001*
HR after 2 min	71.93±11.26	80.27±14.62	0.02*
HR after 3 min	71.07±11.26	80.4±13.18	0.004*
HR after 4 min	72.9±11.17	80.17±14.89	0.037*
HR after 5 min	72.73±9.03	79.23±13.57	0.03*

Physically active males had a lower anticipatory HR. However, when the change in HR (Δ _anticipatory-rest_) is compared, there was no statistically significant difference between physically active and sedentary participants.

The anticipatory and post-exercise heart rate in vigorous-intensity exercise between active and sedentary males is shown in Table 3.

**Table 3:** Anticipatory and post-exercise heart rate in vigorous-intensity exercise ^*^Statistically significant p-value of unpaired t-test ^†^p-value of Mann-Whitney U test HR: Heart rate, Δ_anticipatory-rest_: Difference of anticipatory and resting heart rate

Parameter	Physically active (n = 30)	Sedentary (n = 30)	p-value
Resting HR	76.4±10.89	82.23±12.69	0.06
Anticipatory HR	80.83±11.18	91.7±15.04	0.002*
Δ _anticipatory-rest_	4.43±10.59	9.47±11.48	0.36†
HR immediately after exercise	172.87±15.94	182.53±11.6	0.009*
HR after 1 min	125.6±17.31	146.27±13.2	<0.0001*
HR after 2 min	113.1±17.94	129.4±13.61	0.0002*
HR after 3 min	109.77±15.96	120.16±11.31	0.005*
HR after 4 min	106.97±14.91	115.53±8.85	0.009*
HR after 5 min	104.47±14.41	114.62±10.35	0.005*

Similar to moderate-intensity exercise, physically active individuals had a lower anticipatory heart rate. Similar to the finding in moderate-intensity exercise, in vigorous-intensity exercise, the change in HR (Δ _anticipatory-rest_) showed no statistically significant difference.

## Discussion

The brain's perception of upcoming exercise, influenced by factors like motivation, past experiences, and the perceived intensity of the impending activity, plays a pivotal role in this anticipatory response [12]. We found distinct patterns of anticipatory HR changes between physically active and sedentary individuals during different exercise intensities. While physically active young males showed no significant increase in anticipatory HR in moderate-intensity exercise, sedentary males showed significantly higher anticipatory HR in both exercise protocols indicating a less regulated cardiovascular response. The underlying reason for the finding of no significant change between resting and anticipatory HR may be attributed to the adaptive response of the cardiovascular system. Regular physical activity and exercise training can lead to improved cardiovascular fitness and efficiency, allowing the heart to respond more effectively to the anticipated exercise demand [13]. Research studies have shown that human muscle sympathetic nerve activity and blood flow are modulated during exercise, further supporting the role of the sympathetic nervous system in regulating heart rate [14, 15].

Sedentary individuals may have lower cardiovascular fitness and less efficient heart function, leading to a greater increase in HR during anticipation and exercise. The lack of regular physical activity and exercise training in sedentary individuals may contribute to a less prepared cardiovascular system, resulting in a more pronounced increase in HR in response to more sympathetic activity [16]. This highlights the importance of regular physical activity in improving cardiovascular fitness and promoting a more controlled HR response during exercise.

The underlying reason for the finding of physically active young adult males having significantly lower weight, BMI, and waist circumference compared to sedentary individuals could be attributed to the physiological effects of regular physical activity [17]. Engaging in physical activity promotes energy expenditure, muscle development, and overall metabolic function, leading to a more favourable body composition [18]. However, the lack of significant differences in height, blood pressure, oxygen saturation, and perfusion index suggests that these parameters may be less influenced by physical activity levels and could be influenced by other factors such as genetics or lifestyle choices.

The study has a relatively small sample size, which may limit the generalizability of the findings to larger populations. Another limitation is the lack of diversity in the study population, as the participants consisted only of young adult males. The study relied on self-reported physical activity levels, which may introduce recall bias or subjective interpretation of activity levels. Using objective measures of physical activity, such as accelerometers or fitness trackers, would provide more accurate and reliable data. Furthermore, the study focused on heart rate as the primary cardiovascular parameter. Incorporating a broader range of cardiovascular parameters would provide a more comprehensive understanding of the physiological responses to exercise. The study design was cross-sectional, meaning that it captured data at a single point in time. Longitudinal studies tracking participants over time would provide a more robust analysis of the relationship between physical activity and cardiovascular outcomes.

## Conclusions

This study provides insights into the relationship between physical activity levels and cardiovascular parameters in young adult males. Physically active individuals exhibited lower weight, BMI, and waist circumference compared to sedentary individuals. We found distinct patterns of resting and anticipatory HR changes between physically active and sedentary individuals during different exercise intensities. Physically active young adults showed no significant changes in anticipatory HR before moderate-intensity exercise, suggesting a consistent baseline response. However, they showed an increase in HR before vigorous-intensity exercise. In contrast, sedentary adult males showed significant changes in anticipatory HR indicating a less regulated cardiovascular response. This finding highlights the importance of regular physical activity in modulating HR dynamics and the potential cardiovascular benefits associated with an active lifestyle.
